# Benign and malignant thyroid nodules with autoimmune thyroiditis

**DOI:** 10.20945/2359-3997000000483

**Published:** 2022-06-02

**Authors:** Georgia N. Kassi, Catherine C. Evangelopoulou, Konstantinos D. Papapostolou, Helen J. Karga

**Affiliations:** 1 Alexandra General Hospital Endocrine Unit Athens Greece Endocrine Unit, Alexandra General Hospital, Athens, Greece; 2 Medical Diagnostic Group Athens Greece Medical Diagnostic Group, Athens, Greece

**Keywords:** Thyroid nodules, autoimmune thyroiditis, papillary thyroid cancer

## Abstract

**Objective:**

The prevalence of autoimmune thyroiditis (AT) in papillary thyroid carcinoma (PTC) is still controversial. The aim of this study was to investigate the frequency of coexistence of PTC with AT versus that of the coexistence of benign nodules with AT.

**Materials and methods:**

This was a cross-sectional retrospective study including patients operated on for thyroid nodules from January 2011, to April 2021. The frequency of papillary carcinomas cooccurring with AT was compared to that of benign nodules cooccurring with AT, which was assessed based on cytopathological diagnosis after thyroidectomy.

**Results:**

The study included 668 cases of benign nodules and 420 cases with PTC. No statistically significant difference was observed between cases of benign and PTC nodules regarding the presence of AT (25% vs. 28%, respectively, p = 0.177). The size of the PTC compared to that of the benign predominant nodules was significantly smaller both in the absence (0.96 ± 1.09 cm vs. 2.19 ± 1.06 cm, p < 0.05) and in the presence (0.77 ± 0.76 cm vs. 1.67 ± 1.08 cm, p < 0.01) of AT. In the binary logistic regression analysis of the PTC, the only variable associated with AT was multifocality (odds ratio: 1.750, 95% confidence intervals: 1.131-2.706, p = 0.013). The incidences of lymph node involvement and advanced stage PTC were very low both in the presence and absence of AT.

**Conclusion:**

The nodules present with PTC were not more likely to coexist with AT than benign nodules were. The small incidence of advanced PTC indicates a significant improvement in early-stage diagnosis.

## INTRODUCTION

There is evidence that papillary thyroid carcinoma (PTC) is associated with an increased prevalence of autoimmune thyroiditis (AT), suggesting a link between these two conditions (1,2). However, in some studies, there was no association observed between AT and an increased risk of thyroid cancer (3,4). To date, the data are controversial, and the relationship between AT and PTC remains to be defined (3-6). AT is characterized by diffuse infiltration of the thyroid with lymphocytes and commonly by goiter with solitary or multiple nodules. The majority of these nodules have poor uptake of radioisotopes, raising the possibility of malignancy and supporting the association of PTC and AT (7,8). There is evidence that chronic immune-inflammatory conditions may predispose cells to gene deregulation that drives oncogenic transformation (9). Consequently, AT may be considered a premalignant condition promoting thyroid carcinogenesis (10). Patients who underwent total thyroidectomy for entirely benign indications had an increased prevalence of incidentally discovered PTC in the presence of AT (11). On the other hand, in some studies, similar frequencies of coexistence of both PTC and benign nodules with AT were found (3,4). Furthermore, it is unclear whether the presence of AT is associated with more favorable clinicopathological characteristics and a better outcome in patients with PTC (12,13).

The aim of this study was to investigate the association between benign thyroid nodules and AT versus that of PTC and AT in patients who underwent total thyroidectomy and had a final histological diagnosis. The stage of PTC at the time of diagnosis was also examined.

## MATERIALS AND METHODS

**Figure 1:**
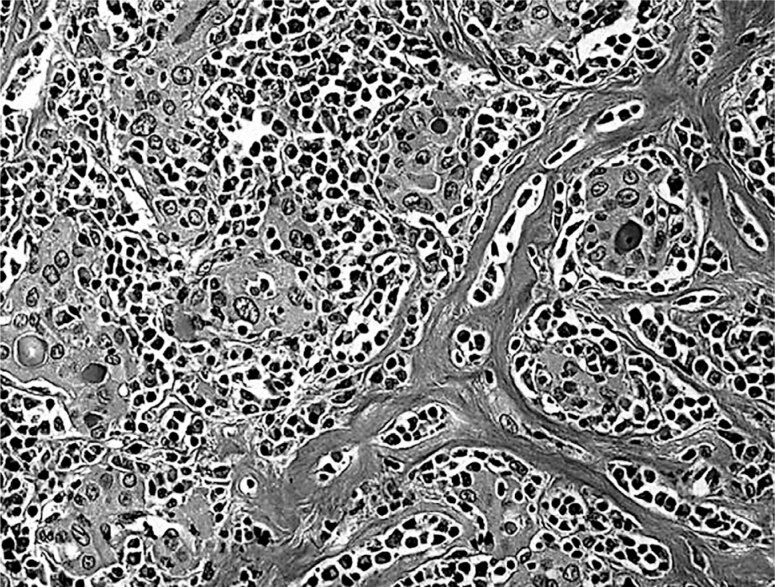
Autoimmune thyroiditis characterized by diffuse thyroid infiltration with lymphocytes and fibrosis.

In this cross-sectional study, the histological results of patients who underwent total thyroidectomy due to nodular goiters between January 2011, and April 2021 were retrospectively evaluated. Patient data were collected by a retrospective review of their medical records. A higher rate of malignancy was expected in this group given that patients received selective operations on nodules with suspicious or malignant cytological findings on biopsy (with or without AT coexistence) or suspicious findings either on thyroid ultrasonography (U/S) or on color flow-Doppler. The cytological findings indicating nodules suspicious for malignancy in the fine needle aspiration biopsy were atypia, Hurthle cell tumor, cellular adenoma, possible low-grade carcinoma and Hashimoto thyroiditis, which many times is difficult to differentiate from lymphoma or carcinoma. The radiological findings indicating nodules suspicious for malignancy in the U/S were taller than wide shape, the presence of microcalcifications, ill-defined margins, high intranodular flow and hypoechogenicity (14). Additionally, patients with rapidly growing nodules underwent surgery. Patients with a history of prior neck radiation exposure, high preoperative serum calcitonin levels and follicular cancer were excluded from the study. The pathologists examined a large number of sections and determined the frequency of small carcinomas. All the slides were reviewed by two independent pathologists with predefined criteria for AT diagnoses, such as widespread lymphocytes, diffuse fibrosis and parenchyma atrophy in both lobes (Figure 1) (15). Focal or peritumoural lymphocytic infiltration surrounding a nodule were not considered AT. For the PTC cases, age, female/male ratio, number of foci, tumor size, extent of local invasion, and distant metastases were estimated, and the final criteria for the histological classification of PTC were kept consistent over time according to the World Health Organization guidelines (16). The most common variants of PTC include conventional PTC, follicular variants, tall cell variants, and other uncommon variants, such as oncocytic, columnar cells, diffuse sclerosing, solid tumors and oxyphilic variants. For the patients with benign lesions, the age, size of the nodules and female/male ratio were recorded. The largest nodule diameter at gross pathological examination was defined as the predominant nodule. The study was approved by the institutional scientific committees of Alexandra General Hospital and Medical Diagnostic Group.

### Statistics

Data analysis was conducted using statistical software (SPSS version 25, Chicago, IL). Continuous data are expressed as the mean ± standard deviation. The Kolmogorov-Smirnov test was applied to assess the normality of variables. Categorical variables are presented as counts and percentages. Comparisons between the analyzed groups were performed by x^2^ test and Fisher's exact test as appropriate and independent-sample t test for data with a normal distribution. A binary logistic regression analysis was performed in cases with PTC to identify variables independently associated with AT. The relative importance of values was presented as odds ratios (ORs) at 95% confidence intervals (CIs). *P* values < 0.05 were considered statistically significant.

## RESULTS

### Comparison between benign and PTC nodules

**Figure 2:**
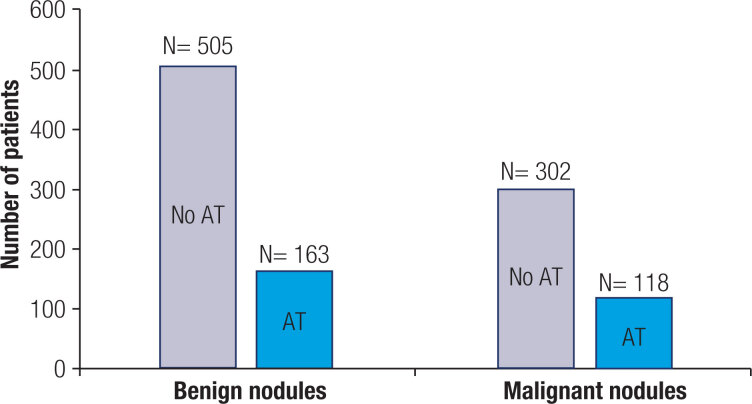
Benign and PTC nodules with or without autoimmune thyroiditis (AT).

Table 1 shows the characteristics and histological findings of the cases with benign and PTC nodules, with the presence or absence of AT. Of the 1088 patients, 668 (61.5%) had benign nodules, and 420 (38.6) had PTC. Of the 668 benign nodules, 163 (24.4%) had clear histological signs of AT involvement, whereas among the 420 PTC nodules, 118 (28%) met the criteria of AT (Figure 2). Using histological type as a constant variable in the calculation, no statistically significant differences were found between benign and PTC nodules regarding the prevalence of AT (OR = 1.211; 95% CI: 0.918-1.596, p = 0.175). Conversely, when AT was the constant variable, although a higher frequency of AT was found in benign nodules than in PTCs (58.5% vs. 41.9%), this difference was not statistically significant (OR = 0.826, 95% CI: 0.627-1.089, p = 0.177). In the absence of AT, the size of the predominant benign nodules, as determined by the initial size estimate, was significantly larger than the size of the PTCs (2.19 ± 1.06 vs. 0.96 ± 1.09, p < 0.05, Table 1). Similarly, in the presence of AT, the size of the benign nodules was significantly larger than that of the PTCs (1.67 ± 1.08 cm vs. 0.77 ± 0.76 cm, p < 0.01). The female/male ratio was significantly different between patients with benign and PTC nodules only when in the presence of AT (p < 0.05, Table 1). Additionally, when in the presence of AT, the size of the benign nodules was significantly smaller than that in the absence of AT (1.67 ± 1.08 cm vs. 2.19 ± 1.06 cm, p < 0.01). There were no significant variations regarding the age at the time of operation between patients with benign or PTC nodules (Table 1).

**Table 1:** Comparisons between benign and PTC nodules

	Benign nodules, n = 668 (61.4%)	PTC nodules, n = 420 (38.6%)	p-value
**Absence of autoimmune thyroiditis**			
No, %	505 (75.6)	302 (71.9)	NS
Age, years	49.2 ± 11.7	49 ± 14.4	NS
Female/male ratio	4.2	3.9	NS
Predominant nodule size, cm	2.19 ± 1.06	0.96 ± 1.09	<0.05
**Presence of autoimmune thyroiditis**			
No, %	163 (24.4)	118 (28.1)	NS
Age, year	45.8 ± 14.1	50.7 ± 11.4	NS
Female/male ratio	11.7	3.8	<0.05
Predominant nodule size, cm	1.67 ± 1.08	0.77 ± 0.76	<0.01

NS: non-significant; PTC: papillary thyroid carcinoma.

### Patients with PTC

Of 420 cases of PTC, based on the predominant cell type, 64% were classified as papillary type (60% conventional, 4% tall cell variant), and 36% were papillary-follicular variants. The same proportion of the different types was found in the cases with or without AT (data not shown). Table 2 shows the characteristics of the PTC patients. Of 420 PTC cases, 118 (28.1%) had clear histological signs of AT involvement, whereas 302 (72%) did not, which is equivalent to 2.5 times more PTC cases without AT. The size of the predominant PTC was significantly larger in the absence than in the presence of AT (0.96 ± 1.09 cm vs. 0.77 ± 0.76 cm, respectively, p < 0.01). When the PTCs were divided into occult cancer (≤1 cm) and carcinomas (>1 cm) by diameter, there was no significant difference between the cases with clear histological signs of AT involvement and those without AT (OR = 1.172, 95% CI: 0.851-1.613, p = 0.337, Table 2). Capsular invasion, lymph node involvement and stage II disease were found to have a very low incidence in cases with or without AT. Most importantly, however, patients with stage III disease (high risk) were very rare, and no cases with distant metastases were identified at the time of operation (Table 2). Based on the binary logistic regression analysis, multifocality, an additional risk factor for PTC recurrence, was observed more frequently with coexisting AT (OR = 1.750, 95% CI: 1.131-2.706, p = 0.013).

**Table 2:** Characteristics of PTC nodules with or without autoimmune thyroiditis

Characteristics		AT	No AT	p-value
Number, (%)		118 (28)	302 (72)	
Age, years		48.5 ± 14.1	50.7 ± 11.4	NS
Female/male ratio		3.9	3.8	NS
Mean size of the largest nodule (cm)		0.77 ± 0.76	0.96 ± 1.09	<0.01
Occult, ≤ 1 cm, (%)		80 (67.7)	219 (72.5)	NS
Multifocality, (%)		53 (44.9)	96 (31.7)	0.013
Capsular invasion, (%)		10 (8.4)	14 (4.5)	NS
Lymph node metastases, (%)		8 (6.7)	14 (4.5)	NS
Clinical stage (8^th^ edition, %)				NS
	I	108 (91.5)	289 (95.7)	
	II	9 (7.6)	12 (3.9)	
	III	1 (0.9)	1 (0.3)	
	Distant metastases	0	0	

PTC: papillary thyroid carcinoma; NS: non-significant; AT: autoimmune thyroiditis; cm: centimeter.

## DISCUSSION

This retrospective study occurring over the last decade shows that the histopathological findings of AT were not differentially distributed between patients with benign nodules and those with PTC.

There are several studies that support a link between PTC and Hashimoto thyroiditis, but such a relationship has not yet been confirmed by other authors and remains an unresolved issue (3,17,18). Findings on the incidence of the coexistence of PTC and AT have a quite large variation across studies, ranging between low (10% and 27%) to almost 58% (19-21). The studies of Erdogan and cols. (5) and Carson and cols. (22) showed a small proportion of PTC in patients with AT. In a recent study of 510 patients with AT, it was found that within 10 years, the appearance of new thyroid nodules was frequent, but none of them were found with differentiated thyroid cancer (23). In an old study, it was suggested that AT is a premalignant thyroid lesion and should have indications for surgery, and it was recommended that patients with AT should have a very close follow-up that could permit an early diagnosis of thyroid cancer (24,25).

The significant differences between the publications could include a variation of the study methods or patients’ inclusion criteria and indications for thyroidectomy (4,26,27). For example, the study of Carson and cols. (22) was performed based on fine-needle aspiration findings, and the study of Castagna and cols. (28) suggested that the association between cancer and AT, found in the histology-based series, was due to a selection bias, since the indication for surgery in the AT group, more frequently than that in the non-AT group, was suspicious cytology. In patients with thyroid nodules, elevated serum levels of antithyroglobulin antibodies and thyroid stimulating hormone ≥1 μIU/ml levels could be independent predictors for PTC (29,30). The differences in environmental factors such as iodine sufficiency between areas play a significant role in the prevalence of AT (31).

Our current study was performed based on the same selection criteria that were used from 1996 to 2011, and the results regarding the prevalence of PTC in AT patients were similar to those of the other study (4). For example, in the present work, the prevalence of coexisting PTC and AT was 28% relative to 31% observed 20 years ago (4). This finding suggests the persistence of lower frequencies of AT in patients with PTC nodules than with benign nodules. It is notable that the sizes of the primary benign or PTC nodules were significantly lower in the presence of AT than without AT. Thus, it could be supported that the autoimmune environment has the potential to recognize either malignant or benign cells and destroy them, inhibiting the rate of progression to large tumors (32). Furthermore, the fact that a significantly smaller size was found in all predominant PTC nodules than in the benign nodules shows that the preoperative diagnostic methods for malignancies have been improved. Additionally, in recent years, an increasing number of patients have self-requested thyroid tests. The above may explain why there were no significant differences in frequencies in the TNM clinical stages or in thyroid capsular invasion in patients with and without AT.

Despite those findings, the higher frequency of multifocality with PTC and thyroiditis should be noted. This finding is in accordance with that of a recent work (33) but in contrast with our previous results (4). Multifocality may be associated with an increased risk of recurrence (34), but we cannot support this finding at present because a longer follow-up may be needed. Furthermore, the regression analysis shows that multifocality was more affected by the presence of AT than the other variables were. It is worth noting that in comparison with our previous report, there were very few patients with thyroid cancer at a high-risk stage, according to criteria of the 8th edition, and no case with distant metastasis was diagnosed at the time of operation.

Possible limitations are the retrospective design of the study and the fact that patients were surgically treated only if nodules were suspicious for malignancy, independent of the preoperative diagnosis of AT. Additionally, a number of patients who did not undergo thyroidectomy may have been missed, particularly those with less evidence of malignancy, such as those with small nodules and potential microcarcinomas (11).

In conclusion, in agreement with our previous work performed over 16 years ago, this latest 10-year study clearly shows that PTC was not associated with an increased prevalence of AT. Consequently, we provide evidence that overtreatment with surgery in cases with cytological findings of thyroiditis and without any other evidence of malignancy could be avoided. Furthermore, the small incidence of severe disease in recent years indicates a significant improvement in the diagnosis of early-stage PTC.
